# Stability of Spline-Type Systems in the Abelian Case

**DOI:** 10.3390/sym10010007

**Published:** 2017-12-27

**Authors:** Darian Onchis, Simone Zappalà

**Affiliations:** 1 Department of Mathematics, University of Vienna, Oskar-Morgenstern-Platz 1, A-1090 Vienna, Austria; 2 Department of Computer Science, West University of Timisoara, Bulevardul Vasile Pârvan 4, 300223 Timisoara, Romania

**Keywords:** spline-type systems, stability, biorthogonal systems, multi-level schemes, constructive realizations, 42C15, 65D15, 42C40

## Abstract

In this paper, the stability of translation-invariant spaces of distributions over locally compact groups is stated as boundedness of synthesis and projection operators. At first, a characterization of the stability of spline-type spaces is given, in the standard sense of the stability for shift-invariant spaces, that is, linear independence characterizes lower boundedness of the synthesis operator in Banach spaces of distributions. The constructive nature of the proof for Theorem 2 enabled us to constructively realize the biorthogonal system of a given one. Then, inspired by the multiresolution analysis and the Lax equivalence for general discretization schemes, we approached the stability of a sequence of spline-type spaces as uniform boundedness of projection operators. Through Theorem 3, we characterize stable sequences of stable spline-type spaces.

## 1 Introduction

Common techniques in signal processing and approximation theory rely on the decomposition of the given sampled functions using shifts of chosen discretized functions possessing optimal localization properties [1–5]. Shift-invariant (SI) spaces are among the standard decomposition tools in approximation and sampling theory [6–8]. Their standard construction [9–11] relies on the two main ingredients: a set of window functions defined over ℝ^*d*^, and the discrete subgroup ℤ^*d*^. SI spaces are built as the closed linear span over *L*^2^(ℝ*^d^*) of the integer shifts of the generating set. SI spaces have been generalized over Locally Compact Abelian (LCA) groups in [12], while in [13] the first two authors have generalized their range function approach [10] to translation-invariant (TI) space on LCA groups to consider the shifts of a countable set of functions over a cocompact subgroup, that is, a subgroup that builds a compact quotient space; this leads again to a SI theory restricted to *L*^2^(ℝ^*d*^).

In this paper we consider a finite set of generators and their shift over cocompact subgroups; the related closure over a selected translation-invariant Banach space will be called spline-type space (ST), as a direct continuation of the notation stated in [6,14,15]. In the standard *L*^2^(ℝ^*d*^) theory [16] for signal analysis, for a countable set of functions Φ ≔ {*ϕ_i_*}_*i*∈*I*_ ⊂ *L*^2^(ℝ^d^) (that are the words of a decomposition vocabulary) the analysis operator *V*_Φ_ and synthesis operator *U*_Φ_ are considered. The first measures through the *L*^2^ product 〈*·*, *·*〉_2_ the presence of each *ϕ_i_* in a given signal: *V*_Φ_*f* ≔ (〈*ϕ_i_*, *f*〉_2_)_*i*∈*I*_ for *f* ∈ *L*^2^ (ℝ^d^); the latter produces a signal starting from a sequence in *L*^2^(*I*): *U*_Φ_*c* ≔ Σ_*i* ∈*I*_ *c_i_ ϕ_i_* for *c* ∈ *L*^2^(*I*). The concept of frames and Riesz basis are countable sets which ensure boundedness of the analysis and synthesis, respectively. In this paper we use the duality principle [17], which has been extended to Banach spaces [18], in its formulation for continuous systems [19] to study the property of ST space through continuous Riesz basis. We develop in this paper the typical computational approach of SI spaces for a general ST space generated by Φ building the biorthogonal Φ̃ system which ensures the reproduction formula:

$$f=\underset{i\in I}{\bar{\sum }}\langle {\tilde{\phi }}_{i},f\rangle \phi_{i},$$

$\bar{\Sigma }$ being the summation over the subgroup that generates the ST space and 〈·, ·〉 the action of a distribution onto a function.

Boundedness of the synthesis operator will be named stability, referring to the literature of SI spaces as boundedness of the synthesis operator and reproduction formula [9,11].

Another aspect is related to the analysis of the projection into ST spaces that could be accounted under the same name stability. To clear this ambiguity comes from our interest of laying the foundations for the stability and the consistency of general discretization schemes [20] in the sense of the Lax–Richtmyer equivalence [21] for the space of multipliers over LCA groups. In the equivalence theorem, stability of discretization method is defined as boundedness of the family of operators, independently from the discretization parameter, that is, uniform boundedness of the family. In analogy with multi-resolution discretization, for example, wavelets systems, we will build a sequence of ST spaces generated by an original ST space and the operator induced by an automorphism over the LCA group, extending the standard construction of the dyadic contraction operator *f*(*x*) ↦ *f* (2*x*) induced by the expansive automorphism *x* ↦ 2*x* over the LCA group ℝ^*d*^ over *L*^2^(ℝ^*d*^).

The outline of the present paper is the following: In Section 2 we introduce basic concepts of locally compact (LC) groups [22–24] to define ST spaces, continuous measurable mappings, p-frames and q-Riesz basis in Banach spaces [19] which correspond to measurable synthesis operators [25]. In Section 3 we generalize in this framework the theory of SI space [11] to obtain a constructive realization of the biorthogoonal system which leads to the non-orthogonal expansion of distributions. Theorem 2 is turned into pseudocode in Algorithm 1 to highlight its computational nature. Finally in Section 4 we introduce the concept of sequence of ST spaces generated by automorphisms of the LCA group, and find the characterization of the induced operators which give stability in the Lax sense.

## 2 Notation and Mathematical Preliminaries

Locally compact (LC) groups are topological groups such that every point has a compact neighbourhood. Notable examples are compact and discrete groups, ℝ^*n*^ and ℚ_*p*_. If the group is Abelian we will shortly say that it is an LCA group. The left translation operator is defined as the operator acting on a function or distribution *f* defined over the LC group *G* as:

$$L_{y}f(x)≔f\left(y^{-1}x\right),\;y,x\in G.$$

A space of function *X* is called translation invariant if *f* ∈ *X* → *L_x_f* ∈ *X* for all *x* ∈ *G*.

The first important property of an LC group is the existence and the uniqueness of the Haar measure, that is, a positive measure invariant under left translation. Lebesgue spaces *L^p^* (*G*) are defined according to this measure, since the standard Lebesgue measure on ℝ^*d*^ coincides with the Haar measure of the LCA additive group ℝ^*d*^. The morphisms from *G* into the torus 𝕋 are called characters of the group. The set of continuous characters of an LC group *G* form together with the multiplication over 𝕋 an LC group *Ĝ* called the topological dual group.

For an LCA group *G*, the topological dual of *Ĝ* is isomorphic to *G*, hence characters *x̂* ∈ *Ĝ* can be represented as *x* ↦ 〈*x*, *x̂*〉 for *x* ∈ *G* and the element of *x ∈ G* as *x̂* ↦ 〈*x*, *x̂*〉, *x̂* ∈ *Ĝ*.

We will use during the paper the same notation for dual of a vector space *V*: given a vector *v* ∈ *V* and a continuous linear functional *w* ∈ V*, the brackets notation 〈*v*, *w*〉 express the application of *w* on *v*.

The Fourier transform of a function in *L*^1^(*G*) is defined as:

$$\hat{f}\left(\hat{x}\right)≔\int_{G}f\left(x\right)\bar{\langle x,\hat{x}\rangle }dx\;\hat{x}\in \hat{G},$$

while the convolution can be defined for the space 𝒦(G) of compactly supported functions:

$$f*g:≔\int_{G}f\left(x\right)L_{x}g\left(y\right)dx$$

and extended to the whole *L*^1^(*G*) as in the case of standard real analysis. It is important to notice that convolution is not commutative for a general LC group. We will denote for a subgroup *H* the convolution *f* *_H_ *g* ≔ ∫*_H_ f*(*x*)*L_x_g*(*y*)*d_H_x*.

Another strength of LCA group theory is the possibility to develop distribution theory. Schwartz class space was generalized by Bruhat for the case of LCA groups by considering them direct limits of elementary LCA groups [26]. It is possible to characterize such a space without the use of smooth structure and differential operators but only by means of the decay property of a function and its Fourier transform [27]: We consider the set 𝒜(G) of functions *f* ∈ *L*^∞^(*G*) for which there exist a compact neighbourhood of the identity *C_f_* ⊂ *G* such that for all *n ∈* ℕ there exist *M_n_ >* 0 such that:

$${\left\|f{|}_{G\backslash C_{f}^{k}}\right\|}_{\infty }\leq M_{n}k^{-n}$$

for all *k ≥* 1. 𝒜(G) is translation invariant, closed under multiplication with *L*^∞^ function, dense in *L*^1^ and a convolution algebra.

The Schwartz–Bruhat space is equivalent to the definition:

$$𝒮\left(G\right)=\left\{f\in 𝒜\left(G\right):\hat{f}\in 𝒜\left(G\right)\right\},$$

and the space of distributions is defined as its dual.

Translation and convolution are weakly extended to distribution in the following way:

$$L_{x}:𝒮{\left(G\right)}^{*}\mapsto 𝒮{\left(G\right)}^{*},\langle f,L_{x}\phi \rangle ≔\langle L_{x^{-1}}f,\phi \rangle ,\forall g\in 𝒮\left(G\right)$$

and

$$*:𝒮\left(G\right)\times 𝒮{\left(G\right)}^{*}\mapsto 𝒮{\left(G\right)}^{*},\langle g,f*\phi \rangle =\langle \tilde{f}*g,\phi \rangle ,\forall g\in 𝒮\left(G\right),$$

where *f*^†^(*x*) = *f* (*x*^−1^). These definitions are well posed also for bounded functions and compactly supported distributions.

The Schwartz–Bruhat space, and its dual, are reflexive space invariant up to translation, character multiplication and convolution with bounded functions.

A consequence of the Paley–Wiener theorem [27] is that the Fourier transform of a compactly supported distribution is a function defined over *Ĝ* with:

$$\langle \langle .,\hat{x}\rangle ,\phi \rangle ={\hat{\phi }}^{†}\left(\hat{x}\right).$$

We introduce spline-type spaces as subspaces of translation-invariant Banach spaces.

**Definition 1.** *Given G an LC group, H a subgroup of G, and* $\Phi ={\left\{\phi_{i}:G\to \mathbb{C}\right\}}_{i=1}^{R}$ *a finite set of functions or distributions in a translation-invariant Banach space* $\left(𝓑,\;{\left||\;\cdot \;|\right|}_{𝓑}\right)$*, the collection of left shift* (Φ, *H*) ≔ *{L_a_ϕ_i_* : *a* ∈ *H, i* = 1,…, *R*} *is called the spline-type system of generating set* Φ *and subgroup H, while its closed span in 𝓑 is called and spline-type space generated by* Φ *and H, which will be indicated as 1D4AE* (Φ, *H*)*.*

In signal analysis, fundamental operators are the analysis and synthesis operators. They are closely related to the vector Lebesgue space over LC group

$$\begin{array}{l} {\left(L^{p}\left(H\right)\right)}^{R}≔\{c\left(a\right)={\left(c_{i}\left(a\right)\right)}_{\underset{a\in H}{i=1,\ldots ,R}}:\\ \;{\left\|c\right\|}_{{\left(L^{p}\left(H\right)\right)}^{I}}≔\underset{i=1,\ldots ,R}{\max}{\left(\int_{H}{\left|c_{i}\left(a\right)\right|}^{p}d\xi \right)}^{\frac{1}{p}}<\infty \}. \end{array}$$

Since we do not restrict our study to discrete sampling, we have to introduce the concept of continuous analysis and synthesis [19]. Given a measure space (Ω, *µ*), a Banach space X and a measurable mapping *F* : Ω → X*, the synthesis of *F* is weakly defined as *U_F_* : *L^q^* (*G*) → X*

$$U_{F}c\left(f\right)≔\int_{H}c\left(\omega \right)\langle f,F\left(\omega \right)\rangle d_{\mu }\omega .$$

For the case of an ST space generated by a a subgroup *H* and a finite set of distributions Φ = (*ϕ_i_*)_*i*=1,…,*R*_, we consider the measure space (Ω, *µ*) ≔ (*H*, *µ_H_*) and *F*(*ω*) ≔ (*L_ω_ϕ_i_*)_*i*=1,…,*R*_ so the synthesis operator has the form:

(1) $$U_{\Phi ,H}c\left(f\right)≔\sum_{i=1}^{R}\int_{H}c_{i}\left(a\right)\langle f,L_{a}\phi_{i}\rangle d_{H}a.$$

Predominant role will have *K*_Φ,*H*_, the kernel of the synthesis of the (Φ, *H*) ST system.

The analysis of a measurable mapping *F* : Ω → X* is the operator *V_F_* : *X* → *L^q^* (*G*) defined as:

$$\begin{array}{ll} V_{F}f(\omega )≔\langle f,F(\omega )\rangle & f\in X,\omega \in \Omega . \end{array}$$

Since we will consider a dual pair of ST spaces, we are interested in mappings *G*Ω → *S* (*G*)** for the reflexive Schwartz–Bruhat space. We will consider analysis in 𝒮*(G) of an ST space generated by $\Phi *={\left(\phi_{i}^{*}\right)}_{i=1,\ldots R}\subset 𝒮(G)**=𝒮(G)$ on a subgroup *H* as:

$$\begin{array}{ll} V_{\Phi ,H}f≔\langle L_{a}\phi_{i}^{*},f\rangle & i=1,\ldots \;,R,a\in H. \end{array}$$

Our definition of ST spaces does not ensure boundedness of these operators between the space of coefficients and the ST space. To introduce the theory of frames and basis, we want to mention the following theorem from ([28], Lemma 3.4.1).

**Theorem 1.** *Consider a bounded linear map T* : *B*_1_ → *B*_2_ *between Banach spaces. If there exist a bounded linear map R* : *B*_2_ → *B*_1_ *such that:*

(2) $$\begin{array}{ll} R\circ Tf=f & for\;all\;f\;in\;a\;dense\;subspace\;of\;B_{1} \end{array}$$

*then*

(I) *R can be extended to T*(*B*_1_)*, i.e., it is a left inverse for T*
(II) *T is a Banach space isomorphism from B*_1_ *onto T*(*B*_1_)*; in particular there exist C*_1_, *C*_2_ *>* 0 *such that:*
  (3) $$\begin{array}{ll} C_{1}{\left\|h\right\|}_{B_{1}}\leq {\left\|Th\right\|}_{B_{2}}\leq C_{2}{\left\|h\right\|}_{B_{1}} & \forall h\in B_{1}. \end{array}$$
(III) *R is surjective and such that:*
  $$\text{if}\;Rh=f\to {\left\|h\right\|}_{B_{2}}\leq C_{2}{\left\|f\right\|}_{B_{1}}.$$
(IV) *P* = *T ○ R is a bounded projection in B*_2_ *onto T*(*B*_1_)*. In particular T*(*B*_1_) *is complementable in B*_2_*, i.e*., ∃*W ⊂ B*_2_ *a closed subspace such that B*_2_ = *T*(*B*_1_) ⊕ *W.*

The strength of the stability theory of linear operator on Banach space relies on the possibility to establish particularly stable algorithms (projections) without the need of working on Hilbert spaces.

Equalities (3) are used in signal analysis over Hilbert spaces to characterize useful dense sets in space of functions [16]: a (not necessarily ([13], Definition 5.1)) countable family of functions in *L*^2^ is called a (continuous) frame if (3) express the boundedness of the *l*^2^ norm of the coefficients obtained through analysis operator by the *L*^2^ norm of the analyzed function; it is called a (continuous) Riesz basis if the inequalities hold for its synthesis. We could extend the Frame–Riesz terminology to a spline-type space, once convergence of the integral over the subgroup is attained, but we prefer to require the more restricting property (2) for the synthesis operator, since standard inequality requirements follow and from property 1 we see that it is the perfect setting for a possible multiresolution approach. We explicitly define frames and bases in *L^p^* Banach spaces.

**Definition 2.** *A weakly measurable mapping F* : Ω → X* *is called a continuous p-frame for X if there exist A*, *B >* 0 *such that:*

$$A\;\left\|\;x\;\right\|\leq {\left(\int_{\Omega }|{\left|\langle x,f\left(\omega \right)\rangle \right|}^{p}d\omega \right)}^{1/p}\leq B\;\left\|\;x\;\right\|.$$

A Bessel mapping is a weakly measurable mapping which ensures the upper bound. The mapping is called a continuous q Riesz basis for X* if 〈*x*, *F*(*ω*)〉 *∀ω ∈* Ω → *x* = 0 *and there exist A*, *B >* 0 *such that:*

(4) $$A{\left\|c\right\|}_{q}\leq \left\|U_{F}c\right\|\leq B{\left\|c\right\|}_{q}\;c\in L^{q}\left(\Omega ,\mu \right).$$

The synthesis operator of *F* on *L^q^* is, for *p* such that $\frac{1}{p}+\frac{1}{q}=1,$ the dual (up to isometric isomorphism from *L^p^* to *L^q^*) of the analysis with value in *L^p^*. Analogous reasoning can be done for the analysis being the dual of the synthesis for a reflexive Banach space *X*.

If *X* is reflexive, then *F* is a continuous p frame if *U_F_* is well defined and bounded, and it has bounds $\left\|{\left(U_{F}^{*}\right)}^{-1}\right\|$ and || *U_F_*|| ([19], Theorem 2.6).

In the representation of signals through a discrete set of functions, central roles have biorthogonal systems [29–31].

**Definition 3.** Given a Banach space X and its dual X*, a biorthogonal system in X × X* is a family ${\left(\phi_{i},\phi_{i}^{*}\right)}_{i\in I}$ *such that* $\langle \phi_{i_{1}},\phi_{i_{2}}^{*}\rangle =\delta_{i_{1},i_{2}}.$

*A biorthogonal system is a projection basis in X*_0_ *⊂ X if it is a basis for X*_0_ *and*

(5) $$P\left(f\right)≔\sum_{i\in I}\langle f,\phi_{i}^{*}\rangle \phi_{i}\;\forall f\in X.$$

*A family is a Riesz projection basis if:*

1. *There is a solid Banach space of coefficients s.t. the synthesis map is a well-defined continuous bijection*.
2. *The synthesis operator has a bounded left inverse*.

In the theory of measurable mappings a continuous p-Bessel mapping *F* : Ω → X* for *X* and a continuous q-Bessel mapping *G* : Ω → X** for X* compose a dual pair (*F*, *G*) if an analogy of (5) holds: For reflexive spaces, the Bessel mappings are dual if the composition of the analysis *V_G_* with the synthesis *U_F_* gives the identity on X* ([19], Lemma 2.4 (ii), Theorem 5.4 (ii) and Definition 5.5). Biorthogonal systems ensure the pair is dual.

For ST systems generated by $\Psi ≔{\left\{\psi_{i}\right\}}_{i=1}^{R}\subset 𝒮\left(G\right)$ and a subgroup *H* we will shortly say that Ψ is biothogonal to $\Phi ≔{\left\{\phi_{i}\right\}}_{i=1}^{R}\subset 𝒮{\left(G\right)}^{*}$ if 〈*L_a_ψ_i_*, *L_b_ϕj*〉 = *δ_i,j_δ_a,b_* for all *i*, *j* = 1,…, *R* and *a*, *b ∈ H*. Thanks to the Haar measure on H, the summation in (5) makes sense for f∈𝒮(G)*.

In the proof of Theorem 2 we will bound from below the synthesis through a constructive procedure which leads to the biorthogonal ST system in *S*(*G*) for the case of a spline-type system in S*(*G*) generated by a compactly supported distribution and a proper subgroup.

Starting from a Bessel system this will lead to the characterization of ST (*L^p^* (*H*))^*R*^-Riesz projection basis for *U*_Φ,*H*_ (*L^p^* (*H*))^*R*^ in the translation-invariant space in which the generating set is selected, and the related left inverse, which also is used in Formula (5), is the analysis with respect to the ST biorthogonal system.

## 3 *L^p^*-Stability of Spline-Type Spaces

In the literature of SI spaces the lower bound expressed in (4) characterizes the injectivity of the synthesis operator and it is called stability of the SI system [11,32,33]. This study has been generalized in *L^p^* (ℝ^*d*^) spaces in [34] but to our knowledge there is no generalization in continuous SI space over LCA groups, that is, ST spaces. In order to introduce the proposed characterization, we show that the theory developed in [32] can be extended to any ST space over LCA group which consider the shifts of a finite set of functions or distributions over an arbitrary cocompact subgroup.

We need to prove first two lemmas; the first characterizes the Fourier transform of a particular synthesized distribution, while the second is a straightforward consequence of the first.

**Lemma 1.** *Let G be an LCA group, H a closed subgroup, ϕ a compactly supported distribution on G, and x̂ ∈ Ĥ.*

*Consider the distribution:*

(6) $$\phi_{\hat{x}}≔\langle \cdot ,\hat{x}\rangle {*}_{H}\phi =U_{\phi ,H}\langle \cdot ,\hat{x}\rangle .$$

*Then ϕ_*x̂*_ defines a linear functional for integrable functions and, for every f ∈ L*^1^(*G*)*, its value can be computed in the dual domain by:*

(7) $$\langle f,\phi_{\hat{x}}\rangle =\int_{H^{⊥}}\hat{\phi }\left(\beta^{-1}\hat{x}\right)\hat{f}\left({\hat{x}}^{-1}\beta \right)d_{H^{⊥}}\beta .$$

**Proof.** Because $𝓐(G){\mathrm{\cdot L}}^{\infty }(G)\mathrm{\subset 𝓐}(G)$, Definition (6) makes sense. Because *ϕ* is continuous on *S* (*G*), and *S* (*G*) dense in *L*^1^(*G*), then for all *f ∈ L*^1^(*G*)

$$\langle f,\phi_{\hat{x}}\rangle =\phi \left(\int_{H}\langle \alpha ,\hat{x}\rangle f\left(\alpha x\right)d_{H}\alpha \right).$$

Fix *x ∈ G* and consider the function:

$$g\left(y\right)=\langle y,\hat{x}\rangle f\left(yx\right)\in L^{1}\left(G\right)$$

as function over *H*. Applying the Poisson’s formula we obtain:

$$\begin{array}{l} \int_{H}g\left(\alpha \right)d_{H}\alpha =\int_{H^{⊥}}\hat{g}\left(\beta \right)d_{H^{⊥}}\beta \\ \;=\int_{H^{⊥}}\langle x,{\hat{x}}^{-1}\beta \rangle \hat{f}\left({\hat{x}}^{-1}\beta \right)d_{H^{⊥}}\beta . \end{array}$$

In this way:

$$\begin{array}{l} \langle f,\phi_{\hat{x}}\rangle =\phi \left(\int_{H^{⊥}}\langle x,{\hat{x}}^{-1}\beta \rangle \hat{f}\left({\hat{x}}^{-1}\beta \right)d_{H^{⊥}}\beta \right)\\ \;=\int_{H^{⊥}}\phi \left(\langle x,{\hat{x}}^{-1}\beta \rangle \right)\hat{f}\left({\hat{x}}^{-1}\beta \right)d_{H^{⊥}}\beta \\ \;=\int_{H^{⊥}}\hat{\phi }\left(\beta^{-1}\hat{x}\right)\hat{f}\left({\hat{x}}^{-1}\beta \right)d_{H^{⊥}}\beta , \end{array}$$

where the last equality is obtained through the definition of the Fourier transform of a distribution.

With the previous lemma we can easily prove whether a character, subsampled to a subgroup *H*, belongs to the kernel *K_ϕ,H_* of the synthesis operator of a principal ST space *S*(*ϕ*, *H*):

**Lemma 2.** *Let G be an LCA group, H a closed subgroup, ϕ a compactly supported distribution on G, then for any x̂ ∈ Ĥ*

$$\langle \cdot ,\hat{x}\rangle \in K_{\phi ,H}\;\text{iff}\;\forall \beta \in H^{⊥},\;\hat{\phi }\left(\beta^{-1}\hat{x}\right)=0.$$

**Proof.** By definition, 〈*·*, *x̂*〉 ∈ *K_ϕ,H_* if and only if *U_ϕ,H_* 〈*·*, *x̂*〉 = 0. Since the hypothesis of Lemma 1 is fulfilled, this is equivalent to saying that the distribution *ϕ_x̂_* built in (6) is an annihilator distribution.

Looking at the right hand side of (7), this can happen if *ϕ̂* (*β*^−1^ *x̂*) = 0 for any *β* ∈ *H^⊥^*.

**Theorem 2.** *Let G be an LCA group, H a cocompact subgroup and a finite generating set $\Phi ={\left\{\phi_{i}\right\}}_{i=1}^{R}$ of compactly supported distributions.*

*There is equivalence* *between*

(i) ∄***f***(*x*) = (*f*_1_(*α*),…, *f_R_*(*α*)) ∈ (*L*^∞^(*H*))^*R*^ *such that U*_Φ,*H*_ ***f*** = 0
(ii) ∄*ξ* ∈ *H*^⊥^ *such that the functions on H*^⊥^, {*ϕ̂_j_* (*·ξ*)}_*j*=1,…,*R*_ *are linearly dependent*
(iii) ∃*δ >* 0 *such that* ∀1 ≤ *p* ≤ ∞, ∀ ***f*** ∈ (*L^p^* (*H*))^*R*^
  (8) $$\delta \;{\left\|f\right\|}_{{\left(L^{p}\left(H\right)\right)}^{R}}\leq {\left\|U_{\Phi ,H}f\right\|}_{L^{p}\left(G\right)}.$$

**Proof.** Theorem 2 (i) → Theorem 2 (ii): If we suppose that ∃*ξ* ∈ *H*^⊥^ and ∃(*a*_1_,…, *a_R_*) *∈* ℂ^*R*^ such that:

$$\phi =\sum_{j=1}^{R}a_{j}{\hat{\phi }}_{j}\left(\beta \xi \right)=0\;\beta \in H^{⊥},$$

then by Lemma 2 〈*·*, *ξ*〉 ∈ *K_ϕ,H_*, so by linearity,

$$\sum_{j=1}^{R}a_{j}\langle \cdot ,\xi \rangle \in K_{\Phi ,H},$$

which contradicts 2.

Theorem 2 (iii) → Theorem 2 (i): It is trivial because if we can find ***f*** ∈ *K*_Φ,*H*_ \ {0}, then *|| **f** ||*_(*L^p^* (*H*))*^R^*_ >|| *U*_Φ,*H*_ ***f*** ||_*L^p^* (*G*)_ = 0.

Theorem 2 (ii) → Theorem 2 (iii): If we consider the correlation matrix:

(9) $$b_{j,k}\left(\alpha \right)\;≔\int_{G}\phi_{j}\left(\alpha x\right)\bar{\phi_{k}\left(x\right)}d_{G}x$$

as function on *H*, because the atoms are compactly supported we can consider the Fourier transform on *H*

(10) $$A_{j,k}\left(\xi \right)=\int_{H}b_{j,k}\left(\alpha \right)\bar{\langle \alpha ,\xi \rangle }d_{H}\alpha \;\xi \in \hat{H}.$$

We prove now that the matrix *A*(*ξ*) is positive definite for every *ξ ∈ Ĥ*.

Because *A*(*ξ*) is trivially Hermitian we only have to prove that:

$$I\left(\xi \right)=\sum_{j,k=1}^{R}a_{j}A_{j,k}\left(\xi \right)\bar{a_{k}}>0\;\forall \left(a_{1},\ldots ,a_{R}\right)\in {\mathbb{C}}^{R}\backslash \left\{0\right\}.$$

Once again, because we are dealing with a finitely generated spline-type scheme, we have to manipulate general combinations of such atoms. Consider then

$$\begin{array}{l} I(\xi )=\int_{H}\sum_{j,k=1}^{R}\left(\int_{G}a_{j}\phi_{j}\left(\alpha x\right)\bar{a_{k}\phi_{K}(x)}d_{G}x\right)\bar{\langle \alpha ,\xi \rangle }d_{H}\alpha \\ \;=\int_{H}\left(\int_{G}\phi \left(\alpha x\right)\bar{\phi (x)}d_{G}x\right)\bar{\langle \alpha ,\xi \rangle }d_{H}\alpha , \end{array}$$

where $\phi =\sum_{j=1}^{R}a_{j}\phi_{j}.$

Now, using the Weil formula and Fubini theorem we obtain:

$$\begin{array}{l} I(\xi )=\int_{H}\left(\int_{G}\phi \left(\alpha x\right)\bar{\phi (x)}d_{G}x\right)\bar{\langle \alpha ,\xi \rangle }d_{H}\alpha \\ \;=\int_{H}\int_{G/H}\int_{H}\phi (\alpha x\gamma )\bar{\phi \left(x\gamma \right)}\bar{\langle \alpha ,\xi \rangle }d_{H}\gamma d_{G/H}\left|x\right|d_{H}\alpha \\ \;=\int_{G/H}\int_{H}\int_{H}\phi (\alpha x\gamma )\bar{\phi \left(x\gamma \right)}\bar{\langle \alpha ,\xi \rangle }d_{H}\gamma d_{H}\alpha d_{G/H}\left|x\right|\\ \;={\int_{G/H}\left|h(x)\right|}^{2}d_{G/H}\left|x\right|, \end{array}$$

where $h\left(x\right):=\int_{H}\phi \left(\alpha x\right)\bar{\langle \alpha ,\xi \rangle }d_{H}\alpha =U_{\phi ,H}\left(\langle .,\xi \rangle \right)\neq 0$ by Lemma 2.

Because it is non-null in the quotient for LCA group too, we have that *I*(*ξ*) is a positive definite.

From this we can compute the inverse of *A*. Since $\text{Φ}={\left\{\phi_{i}\right\}}_{i=1}^{R}$ is a finite generating set of compactly supported distributions, *A* is a matrix in ℂ^*R×R*^ whose entries are trigonometric polynomials, hence each (*A*^−1^)_*j*,*k*_ is a quotient of trigonometric polynomial, whose denominator never vanishes.

Since *H* is cocompact, *H^⊥^* is compact, thus by Wiener’s Lemma, (*A*^−1^)_*j*,*k*_ can be expressed as an absolutely convergent integral in *ℱ*^1^(*H*)

(11) $${\left(A^{-1}\right)}_{j,k}(\xi )=\int_{H}g_{j,k}(\alpha )\bar{\langle \alpha ,\xi \rangle }d_{H}\alpha ,$$

with *g_l,k_* ∈ *L*^1^(*H*).

Considering the vector valued functions on *H*, ***g**_l_* = (*g_l_*_,1_,…, *g_l,R_*), we build the set of functions:

(12) $$\begin{array}{cc} \psi_{1}=U_{\text{Φ,}Hg_{l}} & l=1,\ldots ,R. \end{array}$$

Fixing *k ∈* {1,…, *R*} we consider for all *l* = 1,…, *R* and for every *γ ∈ H* the products:

$$\begin{array}{l} c_{i,j}(\gamma )≔\langle \psi_{l},\phi_{k}\left(\gamma^{-1}.\right)\rangle \\ \;=\sum_{m=1}^{R}\int_{G}\left(\int_{H}g_{l,m}(\alpha )\phi_{m}\left(\alpha^{-1}x\right)\bar{\phi_{k}\left(\gamma^{-1}x\right)}d_{H}\alpha \right)d_{G}x\\ \;=\sum_{m=1}^{R}\int_{H}g_{l,m}\left(\alpha \right)\left(\int_{G}\phi_{m}\left(\alpha^{-1}x\right)\bar{\phi_{k}\left(\gamma^{-1}x\right)}d_{G}x\right)d_{H}\alpha \\ \;=\sum_{m=1}^{R}\int_{H}g_{l,m}\left(\alpha \right)b_{m,k}\left(\alpha^{-1}\gamma \right)d_{H}\alpha , \end{array}$$

hence

$$\begin{array}{l} \int_{H}c_{i,j}\left(\gamma \right)\bar{\langle \gamma ,\xi \rangle }d_{H}\gamma \\ =\sum_{m=1}^{R}\int_{H}\int_{H}g_{l,m}\left(\alpha \right)b_{m,k}\left(\alpha^{-1}\gamma \right)d_{H}\alpha \bar{\langle \gamma ,\xi \rangle }d_{H}\gamma \\ =\sum_{m=1}^{R}A_{m,k}\left(\xi \right){\left(A^{-1}\right)}_{l,m}\left(\xi \right)=\delta_{k,l}. \end{array}$$

This shows that:

$$\langle L_{\gamma }\psi_{l},L_{\alpha }\phi_{k}\rangle =\langle L_{\alpha^{-1}\gamma }\psi_{l},\phi_{k}\rangle =\delta_{\alpha ,\gamma }\delta_{k,l},$$

which means that we have built through the inversion of the matrix *A* in the Fourier domain, a set of atoms Ψ = {*ψ*_1_,…, *ψ_R_*}, which is the biorthogonal system of Φ.

The reproduction formula holds, then ∀ζ∈𝒮(ϕ,H)

$$\zeta =U_{\Phi ,H}f=\sum_{j=1}^{R}\int_{H}f_{j}L_{\alpha }\phi_{j}d_{H}\alpha ,$$

with

$$f_{j}={\left(\langle \zeta ,L_{\alpha }\psi_{j}\rangle \right)}_{\alpha \in H}.$$

If we fix a p-norm, because *ϕ_j_*, *ψ_j_* ∈ *L*^∞^(*G*) for all *j* = 1,…, *R* and *g_jk_* ∈ *L*^1^ for all *j*, *k* = 1,…, *R*, and apply the Young inequality for convolution twice, we obtain:

$$\begin{array}{l} {\left\|f_{j}\right\|}_{L^{p}(H)}\leq {\left\|\zeta \right\|}_{L^{p}(G)}{\left\|\psi_{j}\right\|}_{L^{\infty }(G)}\\ \;\leq {\left\|\zeta \right\|}_{L^{p}(G)}\sum_{m=1}^{R}{\left\|\phi_{m}\right\|}_{L^{\infty }(G)}{\left\|g_{j,m}\right\|}_{L^{1}(G)}. \end{array}$$

Then, we obtain (8) with

$$\delta^{-1}=\underset{j=1,\ldots ,R}{\text{max}}\sum_{m=1}^{R}{\left\|\phi_{m}\right\|}_{L^{\infty }\left(G\right)}{\left\|g_{j,m}\right\|}_{L^{1}\left(G\right)}.$$

The Theorem is particularly appealing since it displays the constructive nature of the theory. We can turn it into Algorithm 1. This algorithm builds the biorthogonal Φ̃ of the given ST system. If the original preserves also an upper bound for the synthesis operator in *L^q^* (*H*) then we obtain the reproduction formula for *f* ∈ X*,

(13) $$f=P_{\Phi ,H}f=U_{\Phi ,H}V_{\tilde{\Phi },H}f.$$

## 4 Stability for Sequence of Projections

In the theory of numerical solutions of PDE, the term stability refers to a property of finite difference equations with increasing finer mesh. Initially coined to express the growth of rounding error, in the Lax theory [21] it has been reformulated as an intrinsic property of the discretization scheme, independent of the particular initial value of the problem. In this paper we consider uniform boundedness of the projection operators into a sequence of ST space obtained by modifying the generating set and the subgroup through a sequence of automorphisms.

One particularly important feature of an automorphism *α* of an LC group is its modulus which is the (unique) positive number Δ(*α*) such that the composition with the Haar measure *µ* on *G* is *α*(*µ*) = Δ(*α*)*µ*. For each automorphism *α* on *G* the adjoint α* is defined as the automorphism on *Ĝ* such that 〈*αx*, *x̂*〉 = 〈*x*, α* *x̂*〉. Modulus, adjoint and inverse of an automorphism satisfy the following properties:

$$\begin{array}{r} \Delta \left(\alpha *\right)=\Delta \left(\alpha \right)\\ \Delta \left(\alpha^{-1}\right)=\Delta {\left(\alpha \right)}^{-1}, \end{array}$$

and composition with an automorphism and Fourier transform follow the property:

$$\hat{f\circ \alpha }=\hat{f}\circ {\left(\alpha *\right)}^{-1}.$$

To build orthonormal wavelets over local field, in [35], expansive automorphism with respect to a subgroup of the additive group structure was introduced. A slightly more restrictive definition is given in [36] through contractive automorphism: The inverse of a contractive automorphism is expansive, but the inverse of an expansive automorphism is not contractive in general. However, both definitions display *scaling property*: an expansive automorphism *σ* has modulus Δ(*σ*) < 1, while a contractive automorphism *τ* has modulus Δ(*τ*) *>* 1.

In the stage of stability we are not interested in contractive nor expansive automorphism; we plan in the future to study how a contractive automorphism induces a multiresolution analysis which provides approximation order as described for SI space in [33]. Our approach resembles the one from [35] since it makes use of automorphisms over the LCA group, but does not require a multiresolution framework. This type of analysis seems to be original in the literature.

We define the operator associated to an automorphism *τ*:

$$D_{\tau }f\text{=}f\circ \tau^{-1}.$$

Immediate properties of the operator *D_τ_* are that (*D_τ_*)^−1^ = *D*_*τ*^−1^_ and that $\left\|D_{\tau }f\right\|=\text{Δ}{\left(\tau \right)}^{\frac{1}{p}}\left\|D_{\tau }f\right\|$ in *L^p^*(*G*) for every 1 ≤ *p* ≤ ∞, that is, the operator is a scalar multiple of an isometric isomorphism.

Similarly as for the shift, we weakly define the D_*,*τ*_ operator for distribution in X* as the adjoint of *D*_τ^−1^_:

$$\langle f\text{,}D_{\text{*,}\tau }\phi \rangle \text{:=}\langle D_{\tau }^{-\text{1}}f\text{,}\phi \rangle \;\forall f\in X\text{.}$$

The definition is well-posed and compatible with the definition of *D_τ_* since for f ∈ X ⊂ X**, $\langle \phi ,D_{**,\tau }f\rangle =\langle D_{*,\tau }^{-1}\phi ,f\rangle =\langle f,D_{*,\tau }^{-1}\phi \rangle =\langle D_{\tau }f,\phi \rangle .$

We now build through *D_τ_* a sequences of projections. Given a vector space of function or distribution *S*, consider the contracted space:

$$S^{\tau }:=\left\{D_{\tau }s\;:\;s\in S\right\}.$$

It is natural to define the sequence:

$$S^{n}:=S^{\tau^{n}}\;n\in \mathbb{N}.$$

*D_τ_* do not commute with shift since:

$$L_{x}D_{\tau }=D_{\tau }L_{\tau^{-1}x}\;D_{\tau }L_{x}=L_{\tau x}D_{\tau },$$

and equivalent properties for D_*,*τ*_. For this reason we also have the following:

**Proposition 1.** *Let* (Φ, *H*) *be a stable ST system of distribution having a biorthogonal system* (Φ̃, *H*)*, and τ an automorphism*.

*Then the contracted space S*(Φ, *H*)*^τ^ is the ST space S*(D_*,*τ*_ Φ, *τH*) *and* (*D_τ_*Φ̃, *τH*) *is the biothogonal dual*.

**Proof.** Applying the commutation law and the definition of D_*,*τ*_ we have for all *x ∈ τH*

$$\begin{array}{r} \langle L_{x}D_{\tau }{\tilde{\phi }}_{i},D_{*\tau }\phi_{j}\rangle =\langle D_{\tau^{-1}}L_{x}D_{\tau }{\tilde{\phi }}_{i},\phi_{j}\rangle \\ \langle L_{\tau^{-1}x}D_{\tau^{-1}}D_{\tau }{\tilde{\phi }}_{i},\phi_{j}\rangle =\langle L_{\tau^{-1}x}{\tilde{\phi }}_{i},\phi_{j}\rangle =\delta_{i,j}\delta_{0,\tau^{-1}x.} \end{array}$$

**Corollary 1.** *The projection operator into the space S*(*D_τ_*Φ, *τH*) *is:*

$$P_{D_{\tau }\text{Φ},\tau H}=U_{D_{\tau }\text{Φ},\tau H}V_{D_{\tau }\tilde{\text{Φ}},\tau H}.$$

The standard dyadic contraction $D_{2}f(x)=\frac{1}{\sqrt{2}}f(2x)$ is a unitary operator over *L*^2^(ℝ^*d*^), for example, 〈*D*_2_*f*, *D*_2_*g*〉 = 〈*f*, *g*〉. The couple (*D_τ_*, D_*,*τ*_) behaves in a similar manner since 〈*D_τ_f*, D_*,*τ*_*ϕ*〉 = 〈*f*, *ϕ*〉, even if the contractions are not isometries. Since these operators are not isometries we are interested in how a frame or a Riesz basis are influenced by such operators.

**Lemma 3.** *Let* (Φ, *H*) ⊂ X a p-frame for X* with frame bounds 0 *< A < B. Then,* (*D_τ_*Φ, *τ H*) *is a p-frame having frame bounds:*

$$0<{\left\|D_{\tau^{-1}}\right\|}^{-1}A<\left\|D_{\tau }\right\|B.$$

*Let* (Φ, *H*) ⊂ X* a q-Riesz basis for X* with bounds 0 *< A < B. Then,* (*D_τ_* Φ, *τH*) *is a q-Riesz basis having bounds:*

$$0<{\left\|D_{\tau }\right\|}^{-1}A<\left\|D_{\tau^{-1}}\right\|B.$$

**Proof.** Let *ψ* ∈ X*; for the analysis operator of (*D_τ_*Φ, *τH*) sums as:

$$\begin{array}{l} \;\sum_{i}\int_{\tau H}|\langle L_{x}D_{\tau }\phi_{i},\psi \rangle {|}^{p}dx\\ =\sum_{i}\int_{\tau H}|\langle D_{\tau }L_{\tau^{-1}x}\phi_{i},\psi \rangle {|}^{p}dx\\ ={\sum_{i}\int_{\tau H}\left|\langle L_{\tau^{-1}x}\phi_{i},D_{*,\tau {-}^{1}}\psi \rangle \right|}^{p}dx\\ ={\sum_{i}\int_{H}\left|\langle L_{x}\phi_{i},D_{*,\tau {-}^{1}}\psi \rangle \right|}^{p}dx, \end{array}$$

hence

$$\begin{array}{c} {\left(\sum_{i}\int_{\tau H}|\langle L_{x}D_{\tau }\phi_{i},\psi \rangle {|}^{p}dx\right)}^{1/p}={\left(\sum_{i}\int_{H}|{\langle L_{x}\phi_{i},D_{*,\tau^{-1}}\psi \rangle |}^{p}dx\right)}^{1/p}\\ \;\leq B{\left\|D_{*,\tau^{-1}}\psi \right\|}_{X*}\leq B\left\|D_{*,\tau^{-1}}\right\|{\left\|\psi \right\|}_{X*}\\ \;\leq B\left\|D_{\tau }\right\|{\left\|\psi \right\|}_{X*}, \end{array}$$

while for the lower bound, consider *ψ* ∈ X*

$${\left\|\psi \right\|}_{X*}={\left\|{\left(D_{\tau }D_{\tau^{-1}}\right)}^{*}\psi \right\|}_{X*}={\left\|D_{\tau^{-1}}^{*}D_{\tau }^{*}\psi \right\|}_{X*}\;\;\leq \|D_{\tau^{-1}}^{*}\|{\left\|D_{\tau }^{*}\psi \right\|}_{X*}\;\;\leq \frac{\left\|D_{\tau^{-1}}\right\|}{A}{\left({\sum_{i}\int_{H}|\langle L_{x}\phi_{i},D_{\tau }^{*}\psi \rangle |}^{p}dx\right)}^{1/p}\;\;=\frac{\left\|D_{\tau^{-1}}\right\|}{A}{\left({\sum_{i}\int_{\tau H}|\langle L_{x}D_{\tau }\phi_{i},\psi \rangle |}^{p}dx\right)}^{1/p}.$$

For the Riesz basis condition we need to consider that for all *f* ∈ *X*

$$\begin{array}{l} \langle f,U_{D_{*,\tau }\Phi ,\tau H}c\rangle =\sum_{i}\int_{\tau H}c\left(x\right)\langle f,L_{x}D_{*\tau }\phi_{i}\rangle dx\\ \;=\sum_{i}\int_{H}c\left(\tau x\right)\langle f,L_{\tau x}D_{*\tau }\phi_{i}\rangle dx\\ \;=\sum_{i}\int_{H}c\left(\tau x\right)\langle f,D_{*\tau }L_{x}\phi_{i}\rangle dx\\ \;=\sum_{i}\int_{H}c\left(\tau x\right)\langle D_{\tau^{-1}}f,L_{x}\phi_{i}\rangle dx\\ \;=\langle D_{\tau^{-1}}f,U_{\Phi ,H}D_{\tau^{-1}}c\rangle \\ \;=\langle f,D_{*\tau }U_{\Phi ,H}D_{\tau^{-1}}c\rangle , \end{array}$$

hence for all *c* ∈ (*L^q^* (*τ H*))^*r*^,

$$\begin{array}{l} \|U_{D_{*,\tau }\Phi ,\tau H}c{\|}_{X*}=\left\|D_{*\tau }U_{\Phi ,H}D_{\tau^{-1}}c\right\|\\ \;\leq \left\|D_{*\tau }\right\|\|U_{\Phi ,H}D_{\tau^{-1}}c{\|}_{X*}\\ \;\leq B\left\|D_{\tau^{-1}}\right\|{\left\|D_{\tau^{-1}}c\right\|}_{{\left(L^{q}\left(H\right)\right)}^{r}}\\ \;\leq B\left\|D_{\tau^{-1}}\right\|{\left\|c\right\|}_{{\left(L^{q}\left(\tau H\right)\right)}^{r}}, \end{array}$$

while for the lower bound, considering *c* ∈ (*L^q^* (*τ H*))^*r*^,

$$\begin{array}{l} {\left\|c\right\|}_{{\left(L^{q}\left(\tau H\right)\right)}^{r}}={\left\|D_{\tau^{-1}}c\right\|}_{{\left(L^{q}\left(H\right)\right)}^{r}}\\ \;\leq \frac{1}{A}\|U_{\Phi ,H}D_{\tau^{-1}}c{\|}_{X*}\\ \;=\frac{1}{A}\|D_{*,\tau^{-1}}D_{*,\tau }U_{\Phi ,H}D_{\tau^{-1}}c{\|}_{X*}\\ \;\leq \frac{1}{A}\left\|D_{*,\tau^{-1}}\right\|\|U_{D_{*,\tau }\Phi ,\tau H}c{\|}_{X*}\\ \;=\frac{\left\|D_{\tau }\right\|}{A}\|U_{D_{*,\tau }\Phi ,\tau H}c{\|}_{X*}. \end{array}$$

**Corollary 2.** *Let* (Ψ, *H*) ⊂ X a p-frame from X* with bounds 0 *< A*_1_ *< B*_1_ *and* (Φ, *H*) ⊂ X* a q-Riesz basis for X* with bounds 0 *< A*_2_ *< B*_2_*. Then for all f* ∈ X*,

$${\left(\left\|D_{\tau^{-1}}\right\|\left\|D_{\tau }\right\|\right)}^{-1}A_{1}A_{2}{\left\|f\right\|}_{X*}\leq \left\|U_{D_{\tau^{n}}\Phi ,\tau^{n}H}V_{D_{\tau^{n}}\Phi ,\tau^{n}H}f\right\|\leq \left\|D_{\tau }\right\|\left\|D_{\tau^{-1}}\right\|B_{1}B_{2}{\left\|f\right\|}_{X*}.$$

The previous corollary gives us a strong constraint for the operator *D_τ_*: For a general bounded and invertible operator *T* on a Banach space, we have that *|| T || || T*^−1^ *||≥* 1 and equality holds only if *T* is a scalar multiple of isometric isomorphism. If strong inequality holds, recursive application of *D_τ_* would let the constant (*|| D*_*τ*^−1^_ *|| || D_τ_ ||*)^−1^ tend to zero and (*|| D*_τ^−1^_ *|| || D_τ_ ||*)^−1^ explode. Since we are interested in uniform boundedness of projection operators we summarise this result in the following theorem.

**Theorem 3.** *For a stable ST space* (Φ, *H*) *with the Riesz bounds A*_2_ *and B*_2_*, having a biorthogonal system with frame bounds A*_1_ *and B*_1_*, the frame bounds of the sequence of projection operators into the spaces S*(*D_τ^n^_* Φ, *τ^n^ H*) *are stable in the following sense:*

$$A_{1}A_{2}{\left\|f\right\|}_{X*}\leq \left\|P_{D_{\tau^{n}}\Phi ,\tau^{n}H}f\right\|\leq B_{1}B_{2}{\left\|f\right\|}_{X*}\;f\in X*,$$

*if D_τ_ is a multiple of an isometric isomorphism.*

**Example 1.** *Let G* = ℤ *and consider the subgroups H_k_* = 2^*k*^ ℤ *for k* ∈ {4, 5, 6, 7, 8} *which can be obtained* *through the automorphism x* ↦ 2X. *The dual of G is* Ĝ=𝕊 *and the orthogonals of H_k_ are the subgroups* $H_{k}^{⊥}=\{e^{i\frac{l}{2^{k}}},l=0,\ldots \;,2^{k}-1\},$ *respectively*.

*As a generating set, consider as a basic tool the standard Hermite cubic basis on* [0, 1]*,*

$$\begin{array}{l} h_{1}(t)=2t^{3}-3t^{2}+1\\ h_{2}(t)=t^{3}-2t^{2}+t\\ h_{3}(t)=-2t^{3}+3*t^{2}\\ h_{4}(t)=t^{3}-t^{2}. \end{array}$$

*At each level k we have considered the functions D*_1/2*^k^*_*h_i_ and sampled over the integers.*

*On each level k the ST system* ({*D*_1/2*^k^*_ *h_i_*, *i* = 1,…, 4*}*, 2*^k^*ℤ) *satisfied the linear independence condition 2 of Theorem 2.*

*In the following table we show the minimum singular value at each level k, we have considered the functions D1/2khi and sampled over the integers in Table 1* *:*

**Remark 1.** *Theorem 3 can be generalized for an arbitrary sequence of automorphisms: given a stable ST space S*(Φ, *H*)*, a sequence of automorphisms* {*τ_n_*}_*n*≥1_, *we have stability for the sequence of ST spaces:*

$$\{\begin{array}{ll} S_{\text{0}}≔S(\Phi ,H) & \\ S_{n}≔S_{n-1}^{\tau_{n}} & n\geq 1 \end{array}$$

*only if a finite number of τ_n_ are not multiples of isometric isomorphisms.*

## 5 Conclusions

In the present work, we proposed and characterized the stability of ST spaces using standard techniques of SI spaces in the more general framework of continuous measurable maps over Banach spaces. The constructive nature of the exposition has been turned into an algorithm that provides a procedure for testing the stability of the input ST system (step 25 in Algorithm 1) and computes the biorthogonal ST system once the test has been passed.

In the subsequent section, we have studied the uniform stability of morphed ST spaces through automorphisms of the LCA group. The approach is original and does not resemble the standard construction of wavelets and shearlets system [37] since no multiresolution constraint is required. We plan to apply these results for the analysis of the stability of Petrov–Galerkin discretization schemes.

## Figures and Tables

**Table 1:** Minimum singular value at each level.

**Level**	*k* = 4	*k* = 5	*k* = 6	*k* = 7	*k* = 8
**Singular value**	0.4299	0.7830	1.4886	2.9000	5.7229

## Box

**Algorithm 1:** Computation of the coefficients as in (12)

**Precondition:** Windows $\Phi ={\left\{\phi_{r}\right\}}_{r=1}^{R},$ subgroup *H*		
1:	**function** COEFFICIENT_COMPUTATION(Φ,*H*)	
2:		
3:	**for** *r* ← 1 to *R* **do**	
4:		
5:	*ϕ̂*_r_ ← *ℱ* (*ϕ*)	
6:		
7:	**end for**	
8:		
9:	**for** *r*, *l* ← 1 to *R* **do**	
10:		
11:	$G_{r,l}\leftarrow {ℱ}^{-1}({\hat{\phi }}_{r}{\hat{\bar{\phi }}}_{l})$	% Convolutions in (9)
12:		
13:	**end for**	
14:		
15:	$G_{r,l}^{\left(s\right)}\leftarrow G_{r,l}(H)$	% Subsampling to *H*
16:		
17:	**for** *r*, *l* ← 1 to *R* **do**	
18:		
19:	${\hat{G}}_{r,l}^{\left(s\right)}\leftarrow {ℱ}_{H}(G_{r,l}^{\left(s\right)})$	% PD matrix in (10)
20:		
21:	**end for**	
22:		
23:	**for** *x* ∈ *H* **do**	
24:		
25:	*ĝ* (*x*) ← (*Ĝ*^(*s*)^ (*x*))^−1^	% Wiener’s inversion in (11)
26:		
27:	**end for**	
28:		
29:	**for** *r*, *l* ← 1 to *R* **do**	
30:		
31:	$g_{r,l}\leftarrow {ℱ}_{H}^{-1}(G_{r,l}^{\left(s\right)})$	% Coefficients (12)
32:		
33:	**end for**	
34:		
35:	**return *g***	
36:		
37:	**end function**	
